# Combined influence of Bt rice and rice dwarf virus on biological parameters of a non-target herbivore, *Nephotettix cincticeps* (Uhler) (Hemiptera: Cicadellidae)

**DOI:** 10.1371/journal.pone.0181258

**Published:** 2017-07-28

**Authors:** Qianjin Wang, Naishun Han, Cong Dang, Zengbin Lu, Fang Wang, Hongwei Yao, Yufa Peng, David Stanley, Gongyin Ye

**Affiliations:** 1 State Key Laboratory of Rice Biology, Ministry of Agriculture Key Lab of Molecular Biology of Crop Pathogens and Insects, Institute of Insect Sciences, Zhejiang University, Hangzhou, China; 2 State Key Laboratory for Biology of Plant Diseases and Insect Pests, Institute of Plant Protection, Chinese Academy of Agricultural Sciences, Beijing, China; 3 USDA/Agricultural Research Service, Biological Control of Insects Research Laboratory, Columbia MO, United States of America; Institut Sophia Agrobiotech, FRANCE

## Abstract

The advent of genetically modified (GM) Bt rice creates the possibility of interactions among Bt crops, crop pathogens and non-target herbivores. In particular, information on how pathogen-infected Bt-expressing plants will influence non-target herbivores is necessary to predict the sustainability of GM cropping systems. Laboratory bioassays were conducted to evaluate the potential combined impacts of rice dwarf virus (RDV) and two Bt rice lines, T1C-19 (Cry1C) and T2A-1 (Cry2A), on non-target green rice leafhopper (GRLH), *Nephotettix cincticeps* (Uhler) (Hemiptera: Cicadellidae). In the first experiment, GRLHs feeding preference tests on Bt rice lines compared to a parental control rice line, MH63, were conducted. As rice plants were uninfected with RDV, GRLHs generally preferred the control MH63 line over the two Bt lines during the initial 8 h, with no significant preference during the following 64 h. As rice plants were infected with RDV, there were no clear preferences between the Bt rice lines and the control MH63 line. In the second experiment, we assessed the combined influence of RDV-infection status and Bt rice lines on GRLH biological parameters. Egg duration, adult weights, and male adult longevity were significantly affected on RDV-infected Bt rice. Other parameters, egg hatching rate, nymph survival and fecundity were not significantly influenced. We infer that interaction effect among two testing Bt rice lines and RDV will not lead to enlarged pest populations, thus demonstrating that growing these two Bt rice lines will poses negligible risk to GRLH in sustainable rice agroecosystems. Long-term field experiments to monitor the population dynamics of GRLHs at large scale need to be carried out to confirm the current results.

## Introduction

Numerous genetically modified (GM) rice lines expressing insecticidal proteins from *Bacillus thuringiensi*s (*Bt*) Berliner have been developed to control lepidopteran pest species [1– 3]. Two Bt rice lines, Huahui 1 and Shanyou 63, both expressing a fused insecticidal protein, Cry1Ab/Cry1Ac, were issued biosafety certificates in 2009 (renewed in 2014) for research into their commercial production in China [3]. However, these two lines have not been approved for commercial production because further and continued assessment of their influence on agroecological and food security risks is necessary.

Although other GM crops have been deployed in many agroecosystems [4, 5], notably decreasing insecticide applications [6], GM rice lines are relatively new and they may pose potential large-scale agricultural risks, including their impact on non-target arthropods, soil ecosystem, the possibility of gene flow, and other unintended effects. These risks have been investigated in GM rice in several laboratory and field experiments, with very little evidence of adverse effects on the agroecology of rice production [2, 3, 7–9]. So far, research has focused on biotrophic interactions between Bt rice and herbivores, or tritrophic interactions among Bt rice and arthropods comprising the major ecological guilds (detritivores, herbivores, and their natural enemies parasitoids and predators). The next step in the on-going analyses of potentially unexpected agricultural risks is based on the understanding that insect herbivores and plant pathogens co-exist within the same rice cropping systems, and are likely to colonize the same rice plant. Herbivores and pathogens rely on rice tissues, and each of these large sets of organisms may alter the suitability and nutritional quality of the host plant for the other set [10–14]. Thus, it is necessary to assess the potential impacts of pathogen-infected Bt rice on non-target arthropods.

The green rice leafhopper (GRLH), *Nephotettix cincticeps* (Uhler) (Hemiptera: Cicadellidae), is an economically important rice pest in Asia [15, 16]. It directly damages rice plants by consuming vascular fluids and causes secondary damage by transmitting viruses and phytoplasma pathogens, particularly the rice dwarf virus (RDV), and rice yellow stunt virus (RYSV) [15– 17]. RDV, a phytoreovirus in the family Reoviridae, is mainly transmitted by GRLH in a persistent-propagative manner [13, 18]. It infects rice plants, causing considerable damage including stunted growth, white chlorotic specks on leaves, delayed and incomplete panicle exsertion [13, 15, 17]. Aside from their influence on rice plants, the longevity of viruliferous GRLHs was prolonged and fecundity decreased relative to non-viruliferous GRLHs [19]. Contrarily, the fecundity of GRLH feeding on virus-infected rice plants was higher when compared to its performance on healthy rice plants [20].

In laboratory assessments, several GRLH ecological fitness parameters, specifically nymphal duration, adult longevity, and fecundity were influenced by Bt rice, which varied according to the expressed insecticidal proteins, Cry1Ab [21], Cry2A or Cry1C [22]. Similarly, field investigations indicated that two types of Bt rice expressing a fused protein, Cry1Ab/Cy1Ac [23, 24], or a single protein, Cry1Ab [21] resulted in a higher GRLH population density compared to the controls. Other Bt rice lines expressing a fused Cry1Ab/Vip3H protein [25], or single Cry2A, or Cry1C proteins [22] showed no significant effects on population density.

As phytopathogens almost certainly infect Bt rice, it is necessary to appraise the potential impacts of pathogen-infected Bt rice on non-target arthropods. Therefore, we hypothesize that pathogens and Bt rice co-existing may influence rice pest biology positively, negatively, or neutrally at the population level, leading to epidemiological levels of pest-associated rice diseases. The outcomes of our analyses will provide new evidence for the influence of Bt rice on agroecology and food security risks. We tested our hypothesis using the RDV and two Bt rice lines, one expressing Cry2A and the other expressing Cry1C, under laboratory conditions.

## Materials and methods

### Leafhopper rearing

The laboratory GRLH colony was founded from individuals collected from the paddy fields at the Zhejiang University experiment farm, Hangzhou, China, in 2014. The paddy fields were set up in 2010 and RDV has never occurred. The colony has been reared and maintained on healthy rice plants (TN1, Taichung Native 1 with GRLH susceptibility at the tillering stage) for 3–4 generations within 80–mesh insect proof cages (50 cm^3^) in a climate chamber at our standard conditions, 27 ± 1°C, 75 ± 5% relative humidity, a 14 L: 10 D photoperiod and light intensity of 3, 500–4, 000 lux.

To obtain RDV-infected GRLH, non-viruliferous nymphs were confined with RDV-infected TN1 rice seedlings (provided by Prof. Li Yi, College of Life Sciences, Peking University, Beijing, China) for 2 days, then transferred to pass the RDV infection along to TN1 rice seedlings. To ensure the GRLHs were viruliferous, nymphs were individually released into separate glass tubes (D = 2.5 cm × H = 25 cm) with one TN1 rice seedling that was given a reference number. Two days later, each rice seedling was separately transplanted in the glasshouse with its reference number and was replaced with new rice seedlings age 15 ± 2 d for the same leafhopper. Ten days later, the GRLHs were individually collected from plants with characteristic RDV symptoms [15]. GRLHs were separately reared on RDV-infected TN1 plants in a glass tube in a separate climate chamber set at our standard conditions. After emergence, one female and one male were mated in an 80-mesh cage (50 cm^3^) with RDV-infected TN1 plants for oviposition. Offspring were collected for RT-PCR as described below. After confirming infections, other offspring were continually reared together to produce a viruliferous colony for the experiments.

### Rice plants

Two Bt rice lines, T1C-19 (Cry1C) and T2A-1 (Cry2A) [26, 27], were used with their non-Bt parental cultivar MH63 as controls. There were two treatments: virus-free and RDV-infected plants. To obtain RDV-infected rice plants, seedlings (age 15 ± 2 d) were exposed to RDV-infected GRLH for 2 days, and then transplanted in the greenhouse for the experiment. The RDV-infected plants were judged by characteristic RDV symptoms [15] and further confirmed by RT-PCR as described below. Virus-free plants were kept separately from RDV-infected GRLH. All plants were cultured in Kimura solution B [28].

### RDV detection by RT-PCR

Fresh rice leaves were homogenized in liquid nitrogen using a mortar and pestle. Total RNA was extracted from separate samples using TRIzol Reagent (Invitrogen, Carlsbad, CA, USA) following the manufacturer’s instructions. cDNA was synthesized from 1 μg of total RNA using TransScript One-Step gDNA Removal and cDNA Synthesis SuperMix (Transgen, Beijing, China). RT-PCR primers were designed based on RDV S8 fragment [29, 30], forward primer, 5’-ATAGCTGGCGTTACGGCTAC-3’; reverse primer, 5’-AAACCGTCCACCTGACTACG-3’. RT-PCR was carried out in a 20 μl reaction containing 10 μl 2 × TransTaq HiFi PCR Super Mix (Transgen, Beijing, China), 1 μl each primer, 3 μl cDNA template, and 5 μl sterile H_2_O. The PCR cycling profile was 94°C for 3 min, 35 cycles of 94°C for 30 sec, 55°C for 30 sec, 72°C for 2 min, and a final extension for 10 min at 72°C. PCR products were separated in 1% agarose gels and stained with ethidium bromide. The presence of viral RNA confirmed infections with three biologically independent replicates for each sample.

### Feeding selection preference

Healthy plants of each rice line were individually planted in separate plastic clay pots (D = 20 cm), randomly arranged in one angle of an equilateral triangle, then covered with a transparent polyethylene-plastic cylinder (D = 18 cm × H = 50 cm), with two side-windows (D = 5 cm) of nylon mesh in the middle part of the cylinder and covered with nylon mesh at the top for ventilation. Twenty virus-free female adults, which had fasted for 2 h, were transferred into each cage. At 2, 4, 8, 24, 48, and 72 h after inoculation, the numbers of insects on each line were counted and recorded. Rice preference was expressed as choice % = the number of GRLHs loading onto a rice line/total numbers of GRLHs loading onto all testing rice lines × 100%, n = 20 independent biological replicates. The experiment was carried out under our standard conditions in a climate chamber.

To measure the feeding selection preference of virus-free insects among RDV-infected plants, the experiment was conducted as just described with RDV-free rice plants.

### Determining biological parameters

The experiment included six treatments, the three rice lines, and two virus infection states. For each treatment, 150 nymphs within 12 h post-hatch were placed individually into the upper chamber of a transparent plastic cage (S1 Fig). The cage was divided into two independent chambers. The upper chamber (D = 10.5 cm × H = 28.5 cm), which had two side-windows (D = 5.2 cm) of copper wire gauze for ventilation and a hole (D = 2.3 cm) covered with a sponge plug, contained a single rice plant. The lower chamber (D = 12.0 cm × H = 6.8 cm) contained rice plant roots and Kimura solution B [28] for plant growth. On the cover of the lower chamber, there was a hole (D = 2.3 cm) for plant growth. The upper chamber was attached to the cover of the lower chamber with two screws. Rice plants were replaced weekly until adult emergence. The survival and developmental status of nymphs were recorded until the adults emerged. The newly emerged adults were weighted individually with a microelectronic balance (AB135-S, Mettler Toledo, Switzerland, the accuracy of 0.01 mg).

After the adults emerged, one female and one male from the same treatment were mated and transferred to a new rearing cage, as previously described. They were allowed to mate and oviposit until the female died. A new male adult was supplemented if the primary male died. The adult survival and number of eggs laid were observed daily. The rice plants were replaced weekly. Numbers of newly hatched nymphs were counted for each treatment. The experiments were conducted in a climate chamber under our standard conditions.

### Statistical analysis

Data on feeding preference were analyzed by a one-factor ANOVA and Duncan’s multiple range test. The impact of rice types, plant RDV-infection status, and their influence on biological performance parameters were analyzed by ANOVA using Proc general linear models (GLM) conducting multiple comparisons; *P* values were sequentially adjusted by the Bonferroni method [22, 31]. All statistical analyses were run using SAS v.9.1 [32].

## Results

### GRLHs feeding selection preference

**Fig 1:**
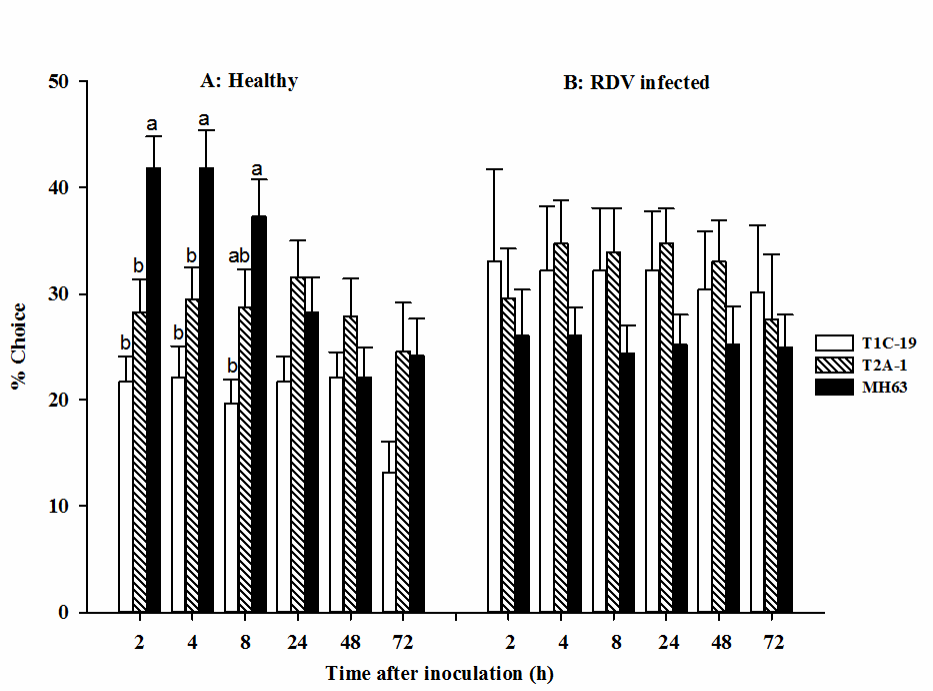
The feeding selection performance of GRLH, *Nephotettix cincticeps* among Bt rice (T1C-19 and T2A-1) along with non-Bt parent control (MH63). (A) Rice plants of each tested line are healthy but not infected by RDV; (B) Rice plants of each tested line are infected by RDV. Columns represent means and bars indicate standard errors of the mean (*n* = 20). At the same time after inoculation by leafhoppers, the columns caped with different low-case letters are significantly different (*P* < 0.05) according to ANOVA based on Duncan’s multiple range tests; otherwise, there are no significant differences.

As rice plants were uninfected with RDV, at initial 2 h and 4 h post-inoculation, the GRLHs preferred the control MH63 over the two Bt rice lines (Fig 1A). By 8 h post-inoculation, there was no difference between MH63 and T2A-1, although the GRLHs preferred these two rice lines over T1C-19. There were no differences between the three lines over the following 64 h (Fig 1A). As rice plants were infected with RDV, the GRLHs showed no significant selection preference among three rice lines (Fig 1B).

### Influence of Bt rice on GRLHs

Among the ten biological performance parameters considered in this work, egg duration (*F* = 36.63, *P* < 0.0001), nymph developmental times (*♂ F* = 43.93, *P* < 0.0001; *♀ F* = 51.24, *P* < 0.0001), adult wet body weights (*♂ F* = 7.50, *P* = 0.0008; *♀ F* = 10.86, *P* < 0.0001), and fecundity (*F* = 6.36, *P* = 0.0031) were significantly different for GRLHs on the Bt lines relative to the control line (Table 1). The Bt lines did not influence the egg hatching rate, nymph survival or adult longevity (Table 1). The egg developmental time was decreased (by 12%) relative to GRLHs on MH63 or GRLHs on T1C-19, but not on T2A-1. Nymphal development time on both Bt lines increased relative to controls for both genders (*♂* by 26%; *♀* by 32%). Adult wet body weights were reduced for both genders and fecundity was reduced on T1C-19 (↓ 38%) and T2A-1 (↓ 42%) (S1 Table).

**Table 1:** Results of multiple comparisons of factors affecting the biological parameters of GRLH.

Parameters	Rice lines		RDV-infected status		Rice lines × RDV-infected status	
	*F*	*P*	*F*	*P*	*F*	*P*
Egg hatching rate (%)	0.42	0.6596	0.01	0.9223	1.24	0.3047
Egg duration (day)	36.63	< 0.0001	17.69	< 0.0001	45.19	< 0.0001
Survival rate of nymphs (%)	2.27	0.1098	18.29	< 0.0001	0.91	0.4048
Total duration of male nymphs (day)	43.93	< 0.0001	0.27	0.6066	0.64	0.5306
Total duration of female nymphs (day)	51.24	< 0.0001	0.53	0.4673	2.06	0.1312
Wet body weight of male adults (mg/adult)	7.50	0.0008	1.62	0.2049	8.95	0.0032
Wet body weight of female adults (mg/adult)	10.86	< 0.0001	2.51	0.1152	3.92	0.0221
Male adult longevity (day)	0.20	0.8197	7.32	0.0089	3.46	0.0378
Female adult longevity (day)	0.89	0.4159	0.21	0.6459	0.50	0.6107
Fecundity (eggs/female)	6.36	0.0031	8.15	0.0059	1.87	0.1636

Parameters were analyzed by general linear models (GLM) using SAS software. When multiple comparisons were conducted, *P* values were sequentially adjusted by the Bonferroni method (adjusted α = 0.0033).

### Influence of RDV-infection status on GRLHs

RDV-infection status significantly influenced the GRLH biological parameters in terms of egg duration (*F* = 17.69, *P* < 0.0001), nymph survival (*F* = 18.29, *P* < 0.0001), male adult longevity (*F* = 7.32, *P* = 0.0089), and fecundity (*F* = 8.15, *P* = 0.0059). Other parameters including egg hatching rate, nymph durations, adult wet body weights and female adult longevity were not influenced by RDV-infection status (Table 1). Developing on RDV-infected rice plants influenced GRLH egg developmental time (↑ 5%), nymphal survival (↓ 50%), male, but not female, longevity (↓ 26%), and fecundity (↓ 35%) (S2 Table).

### Influence of RDV-infected Bt rice plants on GRLHs

RDV-infected Bt rice did not significantly alter the GRLH biological parameters in terms of egg hatch rate, nymph survival, nymph duration, female adult longevity and fecundity. Egg duration (*F* = 45.19, *P* < 0.0001), adult wet body weight (*♂ F* = 8.95, *P* = 0.0032; *♀ F* = 3.92, *P* = 0.0221), and male adult longevity (*F* = 3.46, *P* = 0.0378) were significantly affected by RDV-infected Bt rice (Table 1).

GRLH eggs developed more slowly (by 15%) on T2A-1, but not on T1C-19, compared to control MH63. Development of male nymphs was similar for GRLHs on MN63 and T2A-1 and was reduced in nymphs on T1C-19. Wet body weight of male, but not female adults on Bt rice T1C-19 was reduced compared to GRLHs on control MH63 rice (Table 2).

**Table 2:** The biological parameters of GRLH, *Nephotettix cincticeps* feeding on Bt rice lines (T1C-19 and T2A-1) and non-Bt rice parent control (MH63) plants under the condition of RDV-infection vs. non-infection.

Parameters	T1C-19		T2A-1		MH63	
	Healthy plants	RDV-infected plants	Healthy plants	RDV-infected plants	Healthy plants	RDV-infected plants
Egg hatching rate (%)	97.50 ± 1.12 a (8)	86.67 ± 2.83 a (12)	83.33 ± 6.8 a (10)	100.00 ± 0.00 a (13)	100.00 ± 0.00 a (10)	96.21 ± 1.01 a (14)
Egg duration (day)	6.85 ± 0.12 c (33)	6.08 ± 0.06 d (36)	6.50 ± 0.09 cd (48)	7.90 ± 0.19 a (30)	7.13 ± 0.10 bc (30)	7.67 ± 0.09 ab (46)
Survival rate of nymphs (%)	17.33 ± 7.00 c (15)	56.67 ± 8.03 ab (15)	25.33 ± 7.80 bc (15)	44.67 ± 7.92 abc (15)	38.67 ± 9.30 abc (15)	62.00 ± 6.63 a (15)
Total duration of male nymphs (day)	29.07 ± 1.15 a (14)	28.79 ± 0.56 a (57)	23.88 ± 0.67 b (25)	24.97 ± 0.49 b (37)	22.94 ± 0.42 b (31)	22.93 ± 0.43 b (45)
Total duration of female nymphs (day)	33.83 ± 0.98 a (12)	33.71 ± 1.01 a (28)	26.85 ± 0.78 bc (13)	29.23 ± 0.79 b (30)	26.00 ± 0.50 c (27)	25.23 ± 0.48 c (48)
Wet body weight of male adults (mg/adult)	1.50 ± 0.09 c (12)	1.77 ± 0.04 abc (47)	1.83 ± 0.08 abc (24)	1.71 ± 0.05 b (32)	2.02 ± 0.99 a (26)	1.85 ± 0.05 ab (33)
Wet body weight of female adults (mg/adult)	2.60 ± 0.12 b (11)	2.84 ± 0.07 b (25)	2.98 ± 0.13 ab (13)	2.81 ± 0.09 b (29)	2.98 ± 0.10 ab (25)	3.27 ± 0.05 a (42)
Male adult longevity (day)	21.42 ± 4.43 ab (7)	26.14 ± 2.96 ab (14)	23.10 ± 2.29 ab (10)	23.23 ± 2.37 ab (13)	13.75 ± 2.16 b (8)	29.85 ± 3.52 a (13)
Female adult longevity (day)	23.83 ± 7.14 a (6)	24.69 ± 3.20 a (13)	31.22 ± 4.42 a (9)	25.08 ± 2.72 a (13)	29.40 ± 4.02 a (10)	30.07 ± 3.80 a (14)
Fecundity (eggs/female)	43.88 ± 14.78 b (8)	58.92 ± 11.17 b (12)	41.60 ± 8.49 b (10)	53.92 ± 10.13 b (13)	57.80 ± 8.34 b (10)	107.36 ± 11.04 a (14)

Data are expressed as mean ± standard error, and number of replicates is indicated in parentheses. Means in the same row followed by the same lowercase letters are not significantly different among six treatments based on general linear models (GLM) by Proc GLM (Bonferroni correction, adjusted α = 0.0033).

## Discussion

Our feeding preference study indicated that the GRLHs did not express feeding preferences among the rice lines. First, among three uninfected plants, GRLHs generally preferred the control MH63 line over the two Bt lines during the first 8 h post-inoculation, with no significant preference during the following 64 h. Second, among three RDV-infected plants, we recorded no clear preferences between the Bt rice lines and their parental control MH63 line. With regard to the GRLH rice plant preferences, one study reported that T2A rice did not influence host preference of a target pest, the leaffolder, *Cnaphalocrocis medinalis* Guenée (Lepidoptera: Pyralidae) [33]. Similarly, the T1C-19 rice did not alter the host preferences of the non-target stored product pest, *Rhyzopertha dominica* (F.) (Coleoptera: Bostrychidae), its parasitoid wasp, *Anisopteromalus calandrae* (Howard) (Hymenoptera, Pteromalidae) [34], or the host-searching behavior of another parasitoid, *Cotesia chilonis* (Matsumura) (Hymenoptera: Braconidae) [35]. These findings make sense as T2A-1 and T1C-19 rice had volatile profiles similar to their non-Bt controls [33– 35]. These analyses also help to understand why the GRLHs in this study did not express feeding preferences among the rice lines.

Regarding the factor of Bt rice, we recorded no significant effects of the Bt rice lines on egg hatch, nymphal survival, or adult longevity with the parental control rice line. Egg development was reduced on T1C-19, but not T2A-1. Nymph development was retarded on both Bt rice lines and adult wet weights were reduced. Fecundity, recorded as eggs/female, was substantially reduced on the Bt rice lines compared with the parental control rice line. This is consistent with a previous study which reported longer nymphal duration and lower fecundity of leafhoppers on T1C-19 compared to the controls [22]. Bt rice reduced fecundity of several non-target herbivores such as the brown planthopper, *Nilaparvata lugens* (Stål) (Hemiptera: Delphacidae) [36, 37] and rice thrips, *Stenchaetothrips biformis* (Bagnall) (Thysanoptera: Thripidae) [38]. It is worth noting that there were also some different reports. A higher survival rate of nymphs of GRLH adults fed on T2A-1 Bt rice plants was observed when compared with those on non-Bt rice plants [22]. This situation was quite similar to Zhou et al. [21], where the survival rate of nymphs was higher on transgenic cry1Ab rice (KMD2), similarly, female longevity, and fecundity of GRLHs on transgenic cry1Ab rice (KMD1) were significantly longer/higher than those on wild type variety Xiushui11. In general, the impacts of Bt rice like other Bt crops on non-target pest herbivores may be caused indirectly by unintended alteration of host plants induced by inserted transgenes [39], but not directly by Bt insecticidal proteins [26, 40].

Plant viruses have complex relationships with their insect vectors and host plants [41–44]. Here, RDV-infection status led to increase the GRLH egg duration, nymph survival rate, male adult longevity, and fecundity relative to controls. Other parameters including egg hatch rate, nymph duration, adult wet body weights, and female adult longevity were not influenced by RDV-infection status. RDV-infection influenced nymphal survival and fecundity, consistent with increased GRLH fecundity on RDV-infected rice plants [20]. Our results differ from several reports on the effects of rice viruses on their insect vectors. The fecundity of another leafhopper species, *Recilia dorsalis* Motschulsky (Hemiptera: Deitocephalidae) was reduced on rice infected by Rice gall dwarf virus (RGDV) [45]. Similarly, the females of the white-backed planthopper, *Sogatella furcifera* (Horváth) (Homoptera: Delphacidae) infected by Southern rice black-streaked dwarf virus (SRBSDV) laid fewer eggs compared to non-viruliferous females [46, 47]. These differences may be related to interactions between rice virus species and their respective insect vectors.

Currently, as most studies have focused on biotrophic interactions between Bt rice and herbivores, or interactions between pathogen and herbivores, it is necessary to assess the potential impacts of pathogen-infected Bt rice on non-target arthropods. For the combination effects of Bt rice & RDV-infection status, the current study showed that four parameters, including egg duration, male/female adult wet body weight, and male adult longevity were significantly affected. Other biological parameters, such as egg hatching rate, nymph survival, and fecundity were not influenced by a combination of Bt-rice lines and RDV-infection. The reasons for these differences remain to be unclear. Although these parameters were significantly affected, the effect was numerically small and can be regarded as minor, unlikely to affect the population dynamics of GRLHs in the field considering that other important biological parameters, egg hatching rate, nymph survival, and fecundity were not influenced by the combined Bt-rice lines and RDV-infection. Moreover, Han et al. [48] assessed the current information related to the effects of insect-resistant GM crops on arthropod behavior and drew a conclusion that the majority of reports focused on behavior effects of target insects and the non-target herbivores were rarely affected. Longer egg duration and male adult longevity of the GRLHs on the testing RDV-infected Bt rice lines may slow their population growth, however, we noted that reduced population growth is not a general effect of GM crops [49]. Therefore, we conclude that growing Cry1C- and Cry2A-transgenic rice would pose a negligible risk to the GRLHs. The data reported in this paper bolster our hypothesis that interactions between Bt rice lines and a rice viral disease will not adversely affect fitness of the GRLHs. It is worth noting that although some ecological parameters of non-target herbivores were influenced by Bt rice under laboratory conditions, their density and population dynamics were no different between the Bt and non-Bt rice under field conditions [22, 25]. So long-term and field experiments to monitor the population dynamics of the GRLHs on a large scale need to be conducted to confirm the current results.

## Supporting information

S1 Fig The experimental device for rearing GRLH, *Nephotettix cincticeps* with viruliferous (with white chlorotic specks) or healthy rice seedlings. (TIF)

S1 Table The survival, development and fecundity of GRLH, *Nephotettix cincticeps* feeding on Bt rice lines (T1C-19 and T2A-1) and non-Bt rice parental control (MH63). Data are expressed as mean ± standard error, and number of replicates is indicated in parentheses. In the same row, means followed by the same lowercase letters do not differ significantly based on general linear models (GLM) by Proc GLM (Bonferroni correction, adjusted α = 0.017).
(DOCX)

S2 Table The survival, development and fecundity of GRLH, *Nephotettix cincticeps* feeding on healthy or RDV-infected plants. Data are expressed as mean ± standard error, and number of replicates is indicated in parentheses. In the same row, means followed by the same lowercase letters do not differ significantly based on general linear models (GLM) by Proc GLM (Bonferroni correction, adjusted α = 0.05).
(DOCX)
