# Supplementary figures and images for: Combined influence of Bt rice and rice dwarf virus on biological parameters of a non-target herbivore, Nephotettix cincticeps (Uhler) (Hemiptera: Cicadellidae)
Source: PLoS One. 2017 Jul 28;12(7):e0181258. doi: 10.1371/journal.pone.0181258 (PMC5533439; doi:10.1371/journal.pone.0181258)

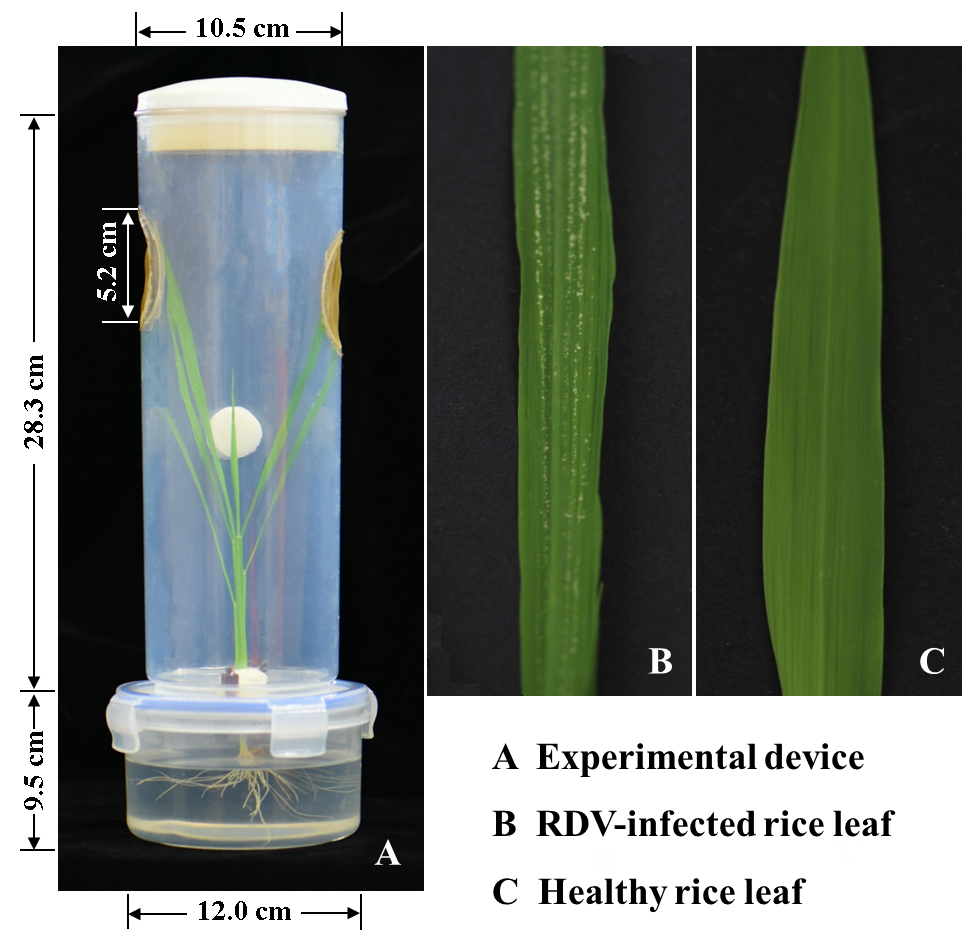

Supplement: S1 Fig — (TIF) [file pone.0181258.s001.tif]
