# Supplementary material for: Combined influence of Bt rice and rice dwarf virus on biological parameters of a non-target herbivore, Nephotettix cincticeps (Uhler) (Hemiptera: Cicadellidae)
Source: PLoS One. 2017 Jul 28;12(7):e0181258. doi: 10.1371/journal.pone.0181258 (PMC5533439; doi:10.1371/journal.pone.0181258)
Supplement: S1 Table — Data are expressed as mean ± standard error, and number of replicates is indicated in parentheses. In the same row, means followed by the same lowercase letters do not differ significantly based on general linear models (GLM) by Proc GLM (Bonferroni correction, adjusted α = 0.017). (DOCX) [file pone.0181258.s002.docx]

Table S1. The survival, development and fecundity of GRLH, ***Nephotettix cincticeps*** feeding on Bt rice lines (T1C-19 and T2A-1) and non-Bt rice parental control (MH63).

| Parameters | T1C-19 | T2A-1 | MH63 | *F* | *P* |
| --- | --- | --- | --- | --- | --- |
| Hatching rate of eggs (%) | 92.08 ± 6.25 a (20) | 91.67 ± 6.59 a (23) | 98.11 ± 5.03 a (24) | 0.42 | 0.6596 |
| Egg duration (day) | 6.47 ± 0.68 b (69) | 7.04 ± 0.08 a (78) | 7.40 ± 0.08 a (76) | 36.63 | < 0.0001 |
| Survival rate of nymphs (%) | 37.00 ± 6.38 a (30) | 35.00 ± 5.75 a (30) | 50.33 ± 6.02 a (30) | 2.27 | 0.1098 |
| Total duration of male nymphs (day) | 28.93 ± 0.51 a (71) | 24.43 ± 0.44 b (62) | 22.93 ± 0.40 c (76) | 43.93 | < 0.0001 |
| Total duration of female nymphs (day) | 33.75 ± 0.66 a (40) | 28.04 ± 0.64 b (43) | 25.62 ± 0.46 c (75) | 51.24 | < 0.0001 |
| Wet body weight of male adults (mg/adult) | 1.65 ± 0.05 b (59) | 1.77 ± 0.05 b (56) | 2.02 ± 0.07 a (59) | 8.59 | 0.0003 |
| Wet body weight of female adults (mg/adult) | 2.72 ± 0.07 b (36) | 2.90 ± 0.07 b (42) | 3.13 ± 0.05 a (67) | 10.86 | < 0.0001 |
| Male adult longevity (day) | 23.79 ± 2.33 a (21) | 23.17 ± 2.11 a (23) | 21.80 ± 2.26 a (21) | 0.20 | 0.8197 |
| Female adult longevity (day) | 24.26 ± 3.18 a (19) | 28.15 ± 2.79 a (22) | 29.74 ± 2.66 a (24) | 0.89 | 0.4159 |
| Fecundity (eggs/female) | 51.40 ± 8.24 b (20) | 47.76 ± 7.60 b (23) | 82.58 ± 7.48 a (24) | 6.36 | 0.0031 |

Data are expressed as mean ± standard error, and number of replicates is indicated in parentheses. In the same row, means followed by the same lowercase letters do not differ significantly based on general linear models (GLM) by Proc GLM (Bonferroni correction, adjusted α = 0.017).
